# The Emerging Role of the c-MET-HGF Axis in Non-small Cell Lung Cancer Tumor Immunology and Immunotherapy

**DOI:** 10.3389/fonc.2020.00054

**Published:** 2020-02-06

**Authors:** Helen F. Titmarsh, Richard O'Connor, Kevin Dhaliwal, Ahsan R. Akram

**Affiliations:** ^1^EPSRC and MRC CDT in Optical Medical Imaging, Universities of Edinburgh and Strathclyde, Edinburgh, United Kingdom; ^2^Centre for Inflammation Research, Queen's Medical Research Institute, University of Edinburgh, Edinburgh Bioquarter, Edinburgh, United Kingdom; ^3^Cancer Research UK Edinburgh Centre, Institute of Genetics and Molecular Medicine, University of Edinburgh, Edinburgh, United Kingdom

**Keywords:** c-MET, HGF, NSCLC, cancer immunology, immunotherapy

## Abstract

Study of the c-Met-HGF axis in non-small cell lung cancer (NSCLC) has focused on the roles of c-MET signaling in neoplastic epithelial cells and the secretion of its ligand hepatocyte growth factor (HGF) by tumor stromal cells. However, there is increasing evidence that some leukocyte sub-sets also express c-MET raising the possibility of an immunomodulatory role for this axis. Consequently, the role of the c-MET- HGF axis in immunoncology is an active area of ongoing research. This review summarizes current knowledge of c-MET expression in NSCLC, the prognostic significance of these findings and the mechanisms by which the c-MET-HGF axis might act in NSCLC, focusing on the emerging evidence for an immunoregulatory role.

## Introduction: Non-Immunological Roles For The c-Met-Hgf Axis In Non-Small Cell Lung Cancer

c-MET is a tyrosine kinase receptor with an extracellular α-chain and a transmembrane β-chain joined by disulphide bonds. In the steady state, the receptor is primarily found on epithelial cells where it regulates cell motility and proliferation (1). The ligand for c-MET is hepatocyte growth factor (HGF), which is produced by mesenchymal cells in an inactive form (1). Activation of HGF requires the action of a serine protease and this occurs in localized in areas of tissue injury (2). Abnormal c-MET signaling has been demonstrated in a number of human cancers as a result of c-MET receptor overexpression, receptor mutation or amplification, HGF overexpression or the formation of abnormal autocrine signaling (3). c-MET activation in cancer promotes; communication between mesenchymal cells and epithelial cells, tissue infiltration, cancer cell proliferation, and the induction of angiogenesis (1, 4–6). In addition selective inhibition or SiRNA knockdown of c-MET decreased the viability of non-small cell lung cancer (NSCLC) cells demonstrating the direct effects of c-MET in promoting tumor growth (7).

In lung adenocarcinomas various missense mutations and alternatively spliced products have been identified. These include Exon-14 splice mutations that result in reduced receptor degradation and prolonged activation of the c-MET receptor, and have been reported to occur in 3% of lung adenocarcinoma and 2.3% other lung tumors (8). *In-vitro* studies by Frampton et al., support the conclusion that Exon-14 splice mutated tumors will respond to anti c-MET therapies. Case reports from three patients also provides limited clinical data to support this conclusion (8). There are also selected reports of clinical responses to c-MET inhibitors in clinical settings in patients with splice mutations or amplification of the c-MET receptor (8–11). The frequency with which human NSCLC tumors express c-MET protein varies between studies, ranging from 25 to 100% and depends on the method of measuring c-MET, as well as and the authors' definition of c-MET positivity (Table 1). The association between tumor production of HGF and NSCLC survival is also summarized (Table 1). These observational studies are limited in their conclusions due to their retrospective nature and it is difficult to directly compare the different methodologies used, however, these studies highlight that there may be a trend toward increased c-MET expression or HGF production with poor patients outcomes. The importance of protein expression and gene amplification has been questioned, as clinical studies using c-MET targeting therapies recruiting patients with protein overexpression or gene amplification have produced disappointing results. This is well-reviewed by Hughes and Siemann (18), although early reports from a phase 1 clinical study of 40 patients [NCT00585195] suggest patients with high MET amplification may respond better to treatment with crizotinib (19). Identifying c-MET pathway activation may be a more appropriate selection criteria (18). With emerging evidence that c-MET may play a role in anti-cancer immune responses, understanding how c-MET may affect immunotherapy is another potential area to explore to see if these therapies could be used to greater effect.

**Table 1:** Studies assessing c-MET and/or HGF in NSCLC, methods of assessment and correlation with survival.

**References**	**Findings**	**Methodology for c-MET or HGF** **assessment**	**Association with survival**
Ichimura et al. (12)	c-MET protein identified in 54% of patients with NSCLC 77% positive in LUAD and 37% positive in LUSC	Immunohistochemistry and western blotting for c-MET receptor in 104 surgical samples	4 year survival times were poorer for adenocarcinoma patients with strong c-met expression in western blot samples compared to patients with c-MET negative adenocarcinomas (*P* < 0.01)
Nakamura et al. (13)	74.6% of adenocarcinomas c-Met positive High expression in 36.1% and phospho-c-Met staining seen in 21.5%. HGF at high levels found in 31.5%	Immunohistochemistry for c-MET, phospho c-MET, and HGF on 130 resected patient lung adenocarcinomas	No relationship seen between MET expression and survival. However, high c-MET expression was associated with metastasis to lymph nodes and advanced pathological stage
Dziadziuszko et al. (14)	25% of samples were c-MET positive using the MetAb scoring system	Immunohistochemistry and/or *in situ* hybridization for MET Gene copy numbers in 189 patients. Assessment of immunohistochemistry samples with two scoring systems. The MetMAb trial scoring system (>50% of cell with moderate or strong staining) and with a hybrid scoring ranging from 0 to 400	No relationship between c-MET protein expression or MET copy number and overall survival was detected
Ma et al. (7)	100% c-MET positive with 61% of NSCLC demonstrating strong c-MET expression (67% LUAD, 57% LUSQ, and 57% large cell strongly positive) Gene overexpression was present in lung adenocarcinoma (adjusted *p* 0.0007) and lung carcinoids (adjusted *p* 0.013). There was no significant difference between gene expression from normal tissues and squamous carcinomas samples (adjusted *p* > 1)	Immunohistochemistry in 32 samples for total c-MET and phospho c-MET. Staining was graded as absent (0), weak (+ 1), or strong (+2)	Survival data not reported in this study
Siegfried et al. (15)	HGF levels not different between patients with stage 1 tumors compared to stage 2 or 3. Higher median and mean concentrations were present in patients where disease reoccurrence was recorded during the follow up period (*p* 0.001)	Quantitative western blot in 56 resected NSCLC samples	Patients with HGF concentrations greater than the median had poorer overall survival (*p* < 0.03). Multivariate cox analysis showed that HGF was an independent prognostic indicator (*p* 0.0001)
Hosoda et al. (16)	High HGF concentrations were present in 32% of patients. No difference in c-Met overexpression in patients in the high and low HGF groups (*p* 0.91)	c-MET expression assessed using immunohistochemistry and c-MET overexpression defined as >50% staining, 10–49% as “middle degree” expression, 1–10% as “minimal” expression and <1% as negative. Whole blood HGF concentrations were measured using an ELSIA quantitative sandwich immunoassay in 25 patients. Patient were divided in to groups with high (>7.2 ng/ml) and low HGF concentrations	Patients with concentrations of HGF > 7.2 ng/ml had a 5 year survival rate of 50% compared to 87.8% of patients in the HGF low HGF (*p* 0.015)
Tsuji et al. (17)	Serum HGF concentrations were raised in patients compared to healthy controls prior to treatment (*p* 0.01)	81 patients with NSCLC with serum HGF levels measured by ELISA. Time points assessed: at pre-treatment, 1–2 months after starting treatment, when best treatment response was recoded and when disease progression occurred	There was a significant association between poor PFS and positive serum HGF 1–2 months after starting treatment *p* < 0.01. In patients with adenocarcinoma treated with second line chemotherapy, high HGF pre-treatment 1–2 months after treatment was associated with poor PFS (*p* < 0.01)

*LUAD, lung adenocarcinoma; LUSC, lung squamous-cell carcinoma; PFS, progression free survival; HGF, hepatocyte growth factor*.

### c-MET and Immune Evasion

The ability to evade anti-tumor immune responses is a hallmark of cancer (20), however the discovery that checkpoint inhibition can overcome immunosuppression has established the anti-cancer potential of adaptive immune responses and ushered in the age of cancer immunotherapy (21). There is evidence that c-MET expression may be associated with expression of immunoregulatory molecules such as programmed cell death ligand (PD-L1) and Indoleamine-2,3-dixoygenase (IDO) in cancer cells. As a result, c-MET presents an attractive therapeutic target as there are clinically available inhibitors which may modulate the ability of cancer cells to evade anti-tumor immune responses. Programmed death 1 (PD-1) is a co-inhibitory molecule expressed on activated T-cells in response to antigenic stimulation. When the activating antigen is cleared PD-1 is down regulated, however where the antigen persists such as in cancer, PD-1 remains highly expressed on T-cells (22). As a means of preventing excessive immune response in the face of chronic antigen stimulation T-cell activity can be suppressed by binding of PD-1 to ligands on dendritic cells, B-cells and macrophage and cancer cells such as PD-L1 which are increased by inflammation or by some cancer mutations (22). PD-L1 binding with PD-1 on activated T-lymphocytes inhibits T-cell proliferation and cytokine release (22–24), thus inhibiting anti-tumor immune responses. PD-L1 expression occurs more frequently with MET activation in NSCLC (25), suggesting that besides its well-characterized direct anti-tumor effects inhibition of c-MET signaling may act indirectly to alleviate immunosuppression. Increasing PD-L1 expression correlated positively with MET gene amplification in 389 NSCLC samples (26). A separate study of 155 resected NSCLC tumor samples found MET activation was associated with PD-L1 expression and demonstrated that in NSCLC cell lines c-MET signaling directly induces PD-L1 expression (25). Aberrant c-MET activity can contribute to acquired tumor cell resistance to treatment with epidermal growth factor receptor (EGFR) targeting tyrosine kinases (TKIs) such as erlotinib (27, 28). This also includes the more recently available, third generation TKI osimertinib (29–31). There is some pre-clinical and clinical evidence that treating patients resistant to EGFR TKIs with c-MET inhibitors may improve outcomes. Treating erlotinib resistant cells with the c-MET inhibitor crizotinib decreased PD-L1 protein and gene expression, demonstrating a positive association between c-MET signaling and PD-L1 expression (32). Clinical reports also document responses to crizotinib in patients with osimertinib resistance (10). In addition, a marked response to a combination of erlotinib and crizotinib given concurrently in a patient with primary erlotinib resistance has also been reported (9). However, it is unknown from patient reports if crizotinib had any effect on PD-L1 expression. The results from Demuth et al. suggest that the c-MET axis may cause resistance to EGFR tyrosine kinases and increase the ability of the tumor to evade immune responses. If a relationship between c-MET inhibitors and PD-L1 expression is found in patient samples, then this could suggest that addition of c-MET inhibitors could also affect tumor immunity. This may be important in tumors with *MET* exon 14 alternations as they response more poorly to immune check point inhibitors. However, in a study where tumor samples with *MET* exon 14-alterations were available for assessment of PD-L1 status, outcomes with PD-1 blockade were poorer than for unselected patients (33). The authors also found treatment outcomes were not better in tumors with higher PD-L1 expression. The impact of the c-MET axis on PD-L1 related outcomes may relate to the type of abnormal c-MET activity present. c-MET may also affect inflammation induced PD-L1 expression.

IFN-γ induced upregulation of PD-L1 expression represents a negative control mechanism to limit the magnitude of T-cell responses (34, 35) and this pathway may also be modulated by c-MET inhibition. In response to IFN-γ produced by anti-tumor T-cells re-invigorated by checkpoint immunotherapy, increased tumor cell expression of PD-L1 can further raise the threshold for effective anti-tumor immunity (36). Although c-MET activation induces PD-L1 expression via a pathway distinct to that used by IFN-γ and independent of JAK/ STAT activation (25), inhibition of c-MET signaling blocks PD-L1 upregulation in response to both HGF and IFN-γ (25, 37). By blocking the capacity of IFN-γ to elevate PD-L1 expression c-MET inhibition may act as an effective co-treatment to increase the effectiveness of immune checkpoint blockade in patients with tumors with aberrant MET activity.

Production of Indoleamine-2,3-dixoygenase (IDO) by tumor cells is another pathway by which the c-MET-HGF axis may contribute to an immunosuppressive tumor microenvironment (38). IDO exerts immunosuppressive effects on T-cells and natural killer cells (NK cells) via regulation of kynurenic pathways and production of metabolites which deplete the extracellular environment of tryptophan required for T-cell function (39). Wang et al. performed experiments using the IDO expressing ovarian cancer cell line SKOV-3. Pharmacological inhibition of the c-MET receptor decreased IDO expression in a concentration dependent fashion. Additionally, when SKOV-3 cells were transfected to express the HGF variant NK4, which is a competitive antagonist for the c-MET receptor, IDO expression was inhibited. The variants expressing NK4 and therefore lacking IDO were more susceptible to the cytotoxic effects of NK cells suggesting that c-MET can indirectly influence NK effects on tumor cells. Murine experiments demonstrated that tumors expressing NK4 recruited more NK cells into the tumor stroma *in-vivo* and had reduced growth compared to tumors in the control group (38). Further work is required to see if increased IDO expression and therefore the function of NK or T-cells can be linked to c-MET expression in NSCLC cells.

The evidence above, indicates that role of aberrant c-MET activity on expression of immunoregulatory molecules by tumor cells is worthy of further investigation in both MET dependent and non-MET dependent NSCLC as is the potential combination of c-MET targeting therapies with immune check point inhibitors.

### Leukocytes and c-MET Expression

**Figure 1:**
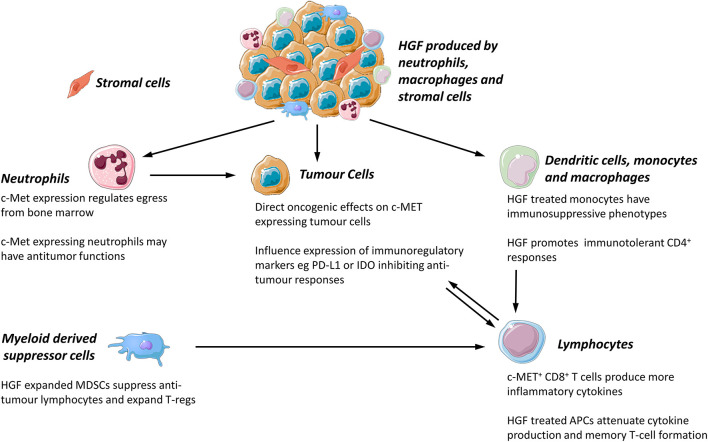
Proposed functions of c-MET/HGF axis in tumor associated leukocyte function. All images were sourced and modified from Servier Medical Art, licensed under a Creative Common Attribution 3.0 Generic License. http://smart.servier.com//.

The expression of c-MET is not limited to epithelial cells. The HGF-c-MET axis is involved in the binding, propagation, and survival of hematopoietic progenitor cells (40) and there is an emerging body of evidence that c-MET is expressed by mature leukocytes in murine and human tissues. The role of c-MET expressing leukocytes in inflammatory, autoimmune, and neoplastic disease is of increasing interest, although the role of these cells in human disease is still incompletely understood. The effect of c-MET on tumor associated leukocytes and cancer outcomes is worthy of further exploration as it may be possible to target leukocytes with c-MET inhibitors. Potential effects of the c-MET-HGF axis on leukocyte functions are summarized in Figure 1.

Neutrophils are best understood for their role within the innate immune system, serving as short lived, phagocytic cells acting against pathogens such as bacteria (41). There is an increasing appreciation of the immunomodulatory role of neutrophils in shaping adaptive immune responses including anti-tumor immunity. The tumor microenvironment influences the phenotype and function of tumor-associated neutrophils (TANs) which can exert both pro- and anti-tumor effects. This has been reviewed elsewhere (42–44). The importance of better understanding the role of neutrophils in lung cancer is clear as they can comprise a significant proportion of the cellular tumor mass, making up between 5 and 25% of the infiltrating leukocytes (45, 46). Importantly neutrophil depletion studies in mice have demonstrated that neutrophils can limit the effectiveness of anti-PD-1 immunotherapy (47). This indicates factors influencing the recruitment and function of neutrophils within the tumor microenvironment may impact the efficacy of checkpoint inhibition (47). Given the use of PD-1 targeting therapies in NSCLC this may be important in the treatment of lung cancer patients. Evidence that c-MET targeting drugs may effect TAN functions and checkpoint inhibition efficacy is summarized below.

Sub-populations of murine and human neutrophils stimulated by inflammatory stimuli express c-MET (48). c-MET is required for the egress of neutrophils from the bone marrow and transendothelial migration of neutrophils into tissues in murine models of cancer (48, 49). c-MET silencing in neutrophils resulted in increased tumor growth in mice, demonstrating c-MET positive neutrophils have anti-tumor properties (48). Furthermore, *in-vitro* experiments showed c-MET knock out neutrophils have reduced nitric oxide-mediated cytotoxicity against neoplastic cells, whereas c-MET positive, HGF-stimulated neutrophils demonstrated increased nitric oxide (NO) release and enhanced cytotoxicity (48). TNF-α produced by tumor cells induced c-MET expression in neutrophils via activation of NF-kB (48), further illustrating how the prevailing cytokine milieu can ignite anti-tumor effector functions in neutrophils. However, besides its beneficial role in tumor cell killing, TAN production of NO may also suppress T-cell responses and limit the effectiveness of immunotherapy. In a murine melanoma model, adoptive T-cell transfer induced the recruitment of immunosuppressive c-MET positive neutrophils to the tumor and tumor draining lymph nodes (49). These TANs displayed increased expression of immunosuppressive genes including PD-L1, ARG1, and iNOS. The majority of TANs expressed PD-L1 and neutrophils from within the tumor and tumor-draining lymph nodes expressed higher levels of c-MET than those found in the spleen (49). Administering c-MET inhibitors alongside adoptive T-cell therapy prevented neutrophil recruitment, improved tumor eradication and increased survival in mouse tumor models (49). Cell-specific knockdown of c-MET expression in neutrophils also enhanced the effectiveness of adoptive T-cell therapy, reducing recruitment of reactive neutrophils and increasing survival of tumor reactive T-cells (49). The different pro and anti-tumor effects of c-Met expressing neutrophils reported in the Glodde and Finisguerra studies may be explained by differences in the inflammatory milieu of the tumor microenvironment. Following either adoptive T -cell therapy or checkpoint inhibition enhanced T-cell responses result in increased IFN-γ production which may promote suppressive effects. PD-L1 expression is readily induced in neutrophils in response to IFN-γ produced by activated T-cells (49). Thus, cytokines produced by both tumor cells [TNF-α promoting c-MET expression (50) and tumor reactive T-cells (via IFN-γ induced PD-L1 expression) (49)] co-operate in driving the recruitment and immunosuppressive phenotype of TANs. Limiting pro-tumor neutrophil mediated T-cell suppression may come at the expense of direct NO-mediated neutrophil killing of tumor cells. Therefore, whether there are anti-cancer benefits to inhibiting the c-MET dependent recruitment of TANs likely depends on the magnitude of the adaptive anti-tumor response. In the presence of a strong anti-tumor immune response or when used as an adjunct to checkpoint blockade or adoptive T-cell therapy, cytotoxic T-cells are likely to be the most efficient anti-tumor effector cells. However, as Glodde et al., where able to demonstrate a positive effect of c-MET silencing and inhibitors in mice with and without c-MET dependent tumors, the possibility of using c-MET inhibitors to improve checkpoint inhibitor associated outcomes in patients is worthy of investigation.

The role of TANs in c-MET associated patient outcomes has been investigated in treatment naive patients with hepatocellular carcinoma (HCC) (51). Although, tumor cell c-MET expression when considered alone was not predictive of outcome, MET expression by HCC cells was inversely associated with overall survival and disease free progression times in the subgroup of patients within this cohort with large numbers of neutrophils at the invading tumor margins (51) Further *in-vitro* work by this group, determined that exposing neutrophils to culture supernatants from HCC cell lines or primary HCC cells increased production of HGF by neutrophils. GM-CSF was found to be increased in culture supernatants from HCC cells and treatment of neutrophils with recombinant GM-CSF lead to increase in HGF, indicating GM-CSF is important in regulating neutrophil production of HGF. The possible pro-tumor effects of these HGF secreting neutrophils was also investigated. Conditioned media from HGF producing TANs promoted hepatoma cell migration, whereas the media from blood derived neutrophils, which produce less HGF did not. In addition, treatment of TANs with c-MET inhibitors reduced TAN associated hepatoma cell migration (51).

The studies by He et al., Finisguerra et al., and Glodde et al., suggest c-MET inhibition could become an adjunct therapy to established immunotherapies because they demonstrate a role for c-MET in the recruitment of TANs, show that tumor derived GM-CSF can stimulate increased HGF-production by neutrophils and that HGF can promote increased expression of PD-L1 and PD-L2 on neutrophils. Further work is needed to see if this could be applicable to the treatment of NSCLC. Currently, few studies have investigated expression of c-MET on TANs from human NSCLC. In four NSCLC patients, where neutrophils were isolated from tumor samples and from non-cancerous lung tissue, gene expression of MET in TANs was greater than in neutrophils from healthy tissues (48). However, in this study there was no assessment of c-MET protein expression or activation. Immunohistochemistry for HGF and the c-MET receptor has been investigated in eight patient bronchioaveolar lavage (BAL) samples and five paired tumor and adjacent non-tumor tissue samples from patients with bronchioaveloar carcinoma (BAC) (52). The c-MET receptor was absent on polymorphonuclear cells suggesting c-MET positive neutrophils are uncommon in BAC. Neutrophils were however a major source of HGF; HGF levels in the BAL fluid correlated with absolute BAL neutrophil counts and increasing BAL HGF concentrations were associated with shorter survival time. BAC, now termed adenocarcinoma spectrum disease, is often a more indolent cancer with a more favorable prognosis. Therefore, care should be taken when applying the results of this study to other types of NCSLC many of which will behave more aggressively.

Lymphocytes play a vital role in influencing immune responses to tumors. Whether lymphocytes mount an immune response or a promote immune tolerance toward tumor cells is influenced by a number of factors including, cytokines, tumor cell immunoregulatory molecules, and secreted proteins, as reviewed by Chraa et al. (53). In addition many recent advancements in NSCLC therapy act to alter T-cell responses. Although to our knowledge c-MET expressing lymphocytes have not be found in human NSCLC cancer there is evidence that subsets of lymphocytes may response to HGF in other types of human cancer. Therefore, if c-MET may have a direct effect on T-cells, potentially augmenting immunotherapy this could provide alternative uses for c-MET inhibitors in both MET dependent and non-MET dependent tumors.

Hepatocyte growth factor can attenuate CD8 positive responses in mice due to effects on antigen presenting cells (APCs)/generating tolerogenic dendritic cells (54, 55). c-Met expression has been identified in human melanoma samples (56) and a small percentage of murine cytotoxic T-cells express c-MET receptors in response to activation (56). c-MET positive CD8 positive T-cells are more cytotoxic than c-MET negative CD8 positive T-cells when assessed *in vivo* and *in vitro* (56). c-MET positive cytolytic T-cells expressed higher levels of granzyme B and perforin and showed a trend toward producing higher concentrations of IFN-γ and TNF-α. However, when these cells were exposed to HGF *in vivo*, cytolytic effects and cytokine production were reduced. This suggests HGF acts to restrain immune responses (56). c-MET inhibition may however, also act directly upon T-cells to enhance effector functions. The hypothesis is supported by experiments that indicate c-MET positive CD8 positive cells have increased ability to reduce melanoma pulmonary metastasis compared to c-MET negative CD8 positive cells, which can be abrogated by the addition of HGF (56). c-MET positive CD8 positive lymphocytes have been identified in human tumors; biopsy samples from four melanoma patients showed 8–35% of CD8 positive T-lymphocytes in close proximity to melanoma cells expressed c-MET (56). The prevalence of c-MET positive T-cells in other cancers, their functional capacities and the consequences of c-MET inhibition on T-cell function requires ongoing research.

c-MET positive B-lymphocytes in solid tumors have not, to the authors knowledge been reported. However, the HGF/c-Met axis has been shown to play a role in survival of mature murine B lymphocytes via the CD74 pathway (57), and is a paracrine factor involved with regulating B cell adhesion in lymphoid tissue (58, 59). High MET mRNA number has been associated with poor outcomes in patients with multiple myeloma (60). Additionally protein expression for c-MET has been found on the plasma cells of 40% of patients with multiple myeloma, but is absent in healthy donors (61). MET expression is documented in 30% of diffuse large B-cell lymphomas in one study (62) and is increased in 73.2% of patients in another study (63). Targeting c-MET has been suggested as a possible therapeutic option for this disease (63). Despite B lymphocytes being present at very low frequencies in NSCLC (46), tumor infiltrating B-lymphocytes have an important impact on CD4+ cells behavior and phenotype (64, 65). Therefore, the possibility that B-cells may express c-MET in the NSCLC microenvironment requires further investigation.

Lymphocyte responses can be influenced by other types of leukocytes such as dendritic cells. As there is some evidence of c-MET expressing dendritic cells targeting these cells may also provide another avenue via which c-MET inhibitors would be used to improve immunotherapy outcomes in cancer patients. Dendritic cells (DCs) are the primary antigen presenting cell population responsible for initiating T-cell activation and development of adaptive immune-responses. c-Met expression in dendritic cells has been best studied in inflammatory disease wherein it drives a tolerogenic rather than immunogenic phenotype. In a model of allergic asthma messenger RNA (mRNA) for c-MET was identified in murine DC cells and HGF suppressed the capability of DC to present antigens (66). HGF has also been shown to promote the development of tolerogenic DCs in experimental autoimmune encephalomyelitis (54). Besides the role of c-MET in promoting immunotolerance, Met-deficient skin DCs failed to migrate to skin draining lymph nodes following activation, resulting in reduced contact hypersensitivity responses (67). These results suggest that c-MET acts on DCs altering adaptive immune responses. Investigating the possible role of the c-MET-HGF axis in the regulation of anti-tumor immune responses is therefore an area worthy of investigation.

Monocytes are circulating precursors to macrophages and dendritic cells, which can have immunomodulatory effects on local T-cell responses (68). c-Met has been found on CD14 positive human monocytes during differentiation and in response to inflammatory cytokines such as lL-6, TNF-α, IFN-γ, IL-1B, and endotoxin (69–71). Activation of monocytes increases secretion of HGF and expression of the enzyme urokinase-type plasminogen activator (uPA) needed to convert pro-HGF to its active form, thus increasing both availability and bioactivity of HGF (70). In addition, HGF increases the transcription of cytokines including IL-6, GM-CSF and G-CSF (70). In contrast Chen et al. found HGF treated monocytes displayed an immunosuppressive phenotype and limited T-cell proliferation in an IL-10 dependent fashion (71). Similarly Rutella et al. found HGF-treated monocytes produced increased amounts of immunosuppressive IL-10 and lower levels of the immunostimulatory cytokine IL-12 p70 (72). Compared to GM-CSF/IL-4 matured monocytes, HGF treated cells were inefficient in driving T-cell proliferation and promoted expansion of suppressive Foxp3 positive regulatory T-cells (72). Gene profiling of HGF stimulated monocytes showed an increase in transcription of genes associated with immunotolerance and tumor progression such as IDO, compared to monocytes stimulated by GM-CSF and IL-4. Co-culturing CD4 positive T-cells and HGF stimulated monocytes with an IDO inhibitor partially reversed the hyporesponsiveness of CD4 positive cells to HGF exposed monocytes. This suggest that HGF may in part exert some immunosuppressive effects by affecting IDO expression. As discussed earlier in this review, the c-MET-HGF pathway has also been implicated in altered IDO expression by tumor cells (38). These studies suggest that HGF/c-MET axis may have indirect suppressive effects on T-cells as a consequence of altering the phenotype of antigen presenting cells favoring tolerogenic rather than immunogenic responses.

Myeloid derived suppressor cells (MDSCs) are a group of leukocytes which act to suppress T-cell effector functions and can be broadly divided into two populations myeloid (M-MDSCs) and polymorphonuclear (PMN-MDSCs) (73). M-MDSCs differ phenotypically from monocytes as they express no or very little of the T-cell ligand HLA-DR and can be recognized as CD11b^+^, HLA-DR^−^, CD14^+^CD15^−^ cells (73). PMN-MDSCs can be classed as CD11b^+^CD15^+^CD14^−/dim^, HLA-DR^−^ cells but are difficult to differentiate from neutrophils (73). Use of these markers as a standard to describe MDSCs has only recently been proposed and therefore literature which describe MDSCS as having a different phenotype are included in this review.

In human blood, CD14 negative leukocytes including MDSCs (CD14^−^, CD11b^+^ CD33^+^) can constitutively express low levels of c-Met (74). Culturing peripheral leukocytes with mesenchymal stem cells (MSCs) or HGF causes an increase in MDSCs. HGF-expanded MDSCs suppress allogeneic lymphocyte proliferation and expressed increased immunosuppressive/cytotoxic enzymes including iNOS and ARG-1 compared to at baseline (74). In addition the MDSCs cells were able to induce T-regs from stimulated peripheral blood leukocytes and the ability of MSCs to expand MDSCs populations was inhibited when c-MET was pharmacologically blocked or if HGF production was silenced in cells (74). This suggests that c-MET may be important in regulation of immunoregulatory processes. Indeed, an abstract from a pre-clinical study of a c-MET targeting therapeutic antibody ARGX-111 indicates that the drug can diminish c-MET positive MDSCs, suggesting a role of this drug in treating c-MET positive tumors and altering c-MET activity of cells in the tumor microenvironment (75).

Overall, there is currently little evidence that c-MET expressing leukocyte subsets exist in NSCLC tumors. However, evidence that the c-MET/HGF axis can influence leukocyte activity in the studies discussed above indicates that c-MET inhibitors may potentially be of benefit via their effects on immune cells. This raises the possibility that c-MET inhibitors could be used in patients with non-MET dependent tumors as well as those with tumor cells with aberrant activity.

## Trials And Investigations Of c-Met Targeting Therapies And Immunotherapy In Met Dependent And Non-Dependent Tumors

The potential for c-MET to influence both immunoregulatory molecules in MET dependent cancers and leukocytes in either MET dependent or MET independent cancer raises the possibility that treatments targeting the c-MET axis could impact on the outcomes of anti-cancer immunotherapies. Current evidence suggest that immune check point inhibitors are less effective in tumors with driver mutations including c-MET (76). The conclusions of a report based on the IMMUNOTARGET registry, suggests where driver mutations are found, targeted therapies such as Tyrosine Kinase Inhibitors should be used before contemplating immunotherapy (76). However, a significant amount of pre-clinical investigation is required before this can be considered and this staged approach does not address the potential for concurrent therapy. The possibility of drug toxicity must also be considered. A clinical studying using crizotinib (prescribed for its effects on ALK rather than c-MET in this incidence), in patients given the PD1- PD-L1 targeting treatment nivolumab resulted in serious hepatic toxicity leading to the end of patient enrollment (77). Drugs and immunotherapies targeting c-MET have been developed for use in people. Early stage human trials using T-cells modified to identify the c-MET and PD-L1 proteins (CAR T-Cells) for the treatment of primary hepatocellular carcinoma are planned (78). The aim of this trial is to determine the efficacy of this therapy in increasing overall survival. The potential for these therapies to alter the immune cell populations as well as targeting neoplastic cells expressing c-MET and PD-L1 could be interesting in light of research suggesting c-MET has immunomodulatory functions. This may allow dual targeting of the c-MET and PD-L1 pathways without risking the toxicities seen when crizotinib was given to patients receiving immunotherapy (77).

The c-MET inhibitor crizotinib has been studied as a means of improving treatment outcomes, via an off target effect. Responses to some chemotherapy agents is partly due to causing “immunogenic cell death” which allows immune cells to be attracted to dying cancer cells and progression free and overall survival have been linked to the expression of receptors associated with immunogenic cell death. Crizotinib has been used to induce immunogenic cell death in a human NSCLC cell line (79). When cells treated with the chemotherapeutic agents and crizotinib were injected into mice more mice remained tumor free after 60 days than mice injected with tumor cells treated with chemotherapy alone. The *in-vitro* and *in-vivo* results suggest the c-MET inhibitor crizotinib increases immunogenic cell death and thus improves treatment outcomes. Additionally, these effects are related to T-lymphocyte infiltration and depletion of T-cells or blockade of IFN-γ abolishes these effects. As crizotinib effects appeared to be off target this again suggests a potential use of c-MET inhibitors in MET dependent and non-dependent tumors.

## Summary

Although, the role of the HGF/c-MET axis as a regulator of immune function is incompletely understood it is emerging as a powerful influence in many aspects of immunity. The studies described above have illustrated its effects on first line responders such as neutrophils, in directing the migration and stimulatory capacity of dendritic cells and monocytes through to directly influencing the function of effector T-cells. These effects occur in concert with, and must be considered alongside, direct effects on tumor cells and stromal cells in the tumor microenvironment. As many novel anti-cancer therapies target immunoregulatory molecules or the c-MET receptor itself understanding the relationship between c-MET and immunological responses to tumors is critical in ensuring these drugs are used to their full potential benefit.

## Author Contributions

HT performed the literature searches and wrote the first draft of the article. HT, RO'C, KD, and AA discussed and contributed to manuscript content. All authors reviewed the manuscript before submission.

### Conflict of Interest

The authors declare that the research was conducted in the absence of any commercial or financial relationships that could be construed as a potential conflict of interest.
