# Corrigendum: The Emerging Role of the c-MET-HGF Axis in Non-small Cell Lung Cancer Tumor Immunology and Immunotherapy

**DOI:** 10.3389/fonc.2020.01516

**Published:** 2020-08-21

**Authors:** Helen F. Titmarsh, Richard O'Connor, Kevin Dhaliwal, Ahsan R. Akram

**Affiliations:** ^1^EPSRC and MRC CDT in Optical Medical Imaging, Universities of Edinburgh and Strathclyde, Edinburgh, United Kingdom; ^2^Centre for Inflammation Research, Queen's Medical Research Institute, University of Edinburgh, Edinburgh Bioquarter, Edinburgh, United Kingdom; ^3^Cancer Research UK Edinburgh Centre, Institute of Genetics and Molecular Medicine, University of Edinburgh, Edinburgh, United Kingdom

**Keywords:** c-MET, HGF, NSCLC, cancer immunology, immunotherapy

In the published article the title was incorrectly given as *The Emerging Role of the c-MET-HGF Axis in Non-small Lung Cancer Tumor Immunology and Immunotherapy*. The correct title is *The Emerging Role of the c-MET-HGF Axis in Non-small Cell Lung Cancer Tumor Immunology and Immunotherapy*.

The authors apologize for this error and state that this does not change the scientific conclusions of the article in any way. The original article has been updated.

